# The Characteristics of the HIV-1 Env Glycoprotein Are Linked With Viral Pathogenesis

**DOI:** 10.3389/fmicb.2022.763039

**Published:** 2022-03-24

**Authors:** Silvia Pérez-Yanes, María Pernas, Silvia Marfil, Romina Cabrera-Rodríguez, Raquel Ortiz, Víctor Urrea, Carla Rovirosa, Judith Estévez-Herrera, Isabel Olivares, Concepción Casado, Cecilio Lopez-Galindez, Julià Blanco, Agustín Valenzuela-Fernández

**Affiliations:** ^1^Unidad de Farmacología, Sección de Medicina, Laboratorio de Inmunología Celular y Viral, Facultad de Ciencias de la Salud de la Universidad de La Laguna (ULL), San Cristóbal de La Laguna, Spain; ^2^Unidad de Virologia Molecular, Laboratorio de Referencia e Investigación en Retrovirus, Centro Nacional de Microbiologia, Instituto de Salud Carlos III, Madrid, Spain; ^3^Institut de Recerca de la Sida IrsiCaixa, Institut d’Investigació en Ciències de la Salut Germans Trias i Pujol (IGTP), Barcelona, Spain; ^4^Chair of Infectious Diseases and Immunity, Faculty of Medicine, Universitat de Vic-Universitat Central de Catalunya (UVic-UCC), Barcelona, Spain

**Keywords:** HIV, viral envelope, functional defect, elite controllers (HIV-1 EC), functional envelope, lack of virological control

## Abstract

The understanding of HIV-1 pathogenesis and clinical progression is incomplete due to the variable contribution of host, immune, and viral factors. The involvement of viral factors has been investigated in extreme clinical phenotypes from rapid progressors to long-term non-progressors (LTNPs). Among HIV-1 proteins, the envelope glycoprotein complex (Env) has been concentrated on in many studies for its important role in the immune response and in the first steps of viral replication. In this study, we analyzed the contribution of 41 Envs from 24 patients with different clinical progression rates and viral loads (VLs), LTNP-Elite Controllers (LTNP-ECs); Viremic LTNPs (vLTNPs), and non-controller individuals contemporary to LTNPs or recent, named Old and Modern progressors. We studied the Env expression, the fusion and cell-to-cell transfer capacities, as well as viral infectivity. The sequence and phylogenetic analysis of Envs were also performed. In every functional characteristic, the Envs from subjects with viral control (LTNP-ECs and vLTNPs) showed significant lower performance compared to those from the progressor individuals (Old and Modern). Regarding sequence analysis, the variable loops of the gp120 subunit of the Env (i.e., V2, V4, and mainly V5) of the progressor individuals showed longer and more glycosylated sequences than controller subjects. Therefore, HIV-1 Envs from virus of patients presenting viremic control and the non-progressor clinical phenotype showed poor viral functions and shorter sequences, whereas functional Envs were associated with virus of patients lacking virological control and with progressor clinical phenotypes. These correlations support the role of Env genotypic and phenotypic characteristics in the *in vivo* HIV-1 infection and pathogenesis.

## Introduction

Pathogenesis of viral infections is the result of complex interactions between host genetics, immune responses, and viral factors. In human immunodeficiency virus type 1 (HIV-1) infection and pathogenesis, the role of host (Migueles et al., 2000; Fellay et al., 2009; Miura et al., 2009; Buckheit et al., 2012a; Migueles and Connors, 2015; Naranbhai and Carrington, 2017), immune (Goulder et al., 1997; Deeks and Walker, 2007; Migueles et al., 2008; Buckheit et al., 2012a,b; Carrington and Walker, 2012; Pernas et al., 2012; Martin-Gayo et al., 2015; Cortes et al., 2018; Goulder and Deeks, 2018), and viral factors (Lassen et al., 2009; Blankson, 2010; Casado et al., 2013, 2018; Cabrera-Rodriguez et al., 2019) have been widely investigated. The interactions of these factors have been primarily studied in extreme clinical phenotypes like rapid progressors (RPs) (Dalmau et al., 2009; Jarrin et al., 2015) or long-term non-progressors (LTNPs), LTNP-Elite Controllers (LTNP-ECs), HIV controllers, or Elite suppressors (ES) (Casado et al., 2013, 2018, 2020; Cabrera-Rodriguez et al., 2019; Lopez-Galindez et al., 2019).

Due to these entangled interactions, the investigation of the role of viral proteins and their specific properties in HIV-1 pathogenesis is challenging. Among the viral proteins, the envelope glycoprotein complex (Env) has attracted numerous studies because of its essential role in the immune response and in the initial events of the HIV-1 biological cycle (Miedema et al., 1994; Checkley et al., 2011; Engelman and Cherepanov, 2012; Berger, 2015; Beitari et al., 2019), i.e., the binding to the cellular receptors (Dalgleish et al., 1984; Klatzmann et al., 1984; Alkhatib et al., 1996; Choe et al., 1996; Deng et al., 1996; Feng et al., 1996; Oberlin et al., 1996; Chan and Kim, 1998; Wyatt and Sodroski, 1998; Blanco et al., 2000; Engelman and Cherepanov, 2012; Colin et al., 2013; Garcia-Perez et al., 2015; Herschhorn et al., 2017). The binding efficiency of the viral Env to the CD4 receptor determines further steps of the viral cycle: virus-cell signaling, fusion, and cell-to-cell virus transfer capabilities (Valenzuela-Fernandez et al., 2005; Casado et al., 2018; Cabrera-Rodriguez et al., 2019). HIV-1 Envs unable to stabilize microtubules (i.e., increasing post-transductional acetylation of Lys^40^ residue in α-tubulin), to reorganize F-actin for the delineation of pseudopod-entry virus hot zones present low CD4 binding, restricted fusion, and low early infection (Valenzuela-Fernandez et al., 2005; Barrero-Villar et al., 2009; Garcia-Exposito et al., 2013; Casado et al., 2018; Cabrera-Rodriguez et al., 2019).

There are few reports investigating the characteristics of viral Envs from HIV individuals with different clinical characteristics. Lassen et al. studied the entry efficiency of viral Envs from ES individuals relative to chronically infected viremic and chronic progressors. Envs from ES showed decreased entry efficacy and slower entry kinetics than those of chronic progressors (Lassen et al., 2009). Our group studied the CD4 binding, signaling capacity, and fusogenicity of viral Envs from viremic non-progressors (VNPs) that were similar to those of progressors individuals (Cabrera-Rodriguez et al., 2019). In previous reports, deficient viral Env glycoproteins, because of poor CD4 binding, low transfer, and signaling capacity (Casado et al., 2018), were identified in a cluster of poor replicating viruses from a group of LTNP-ECs without clinical progression for more than 20 years (Casado et al., 2013, 2018). Thus, these works have established that viral Env plays an important role in the pathogenesis control in LTNPs (Marconi et al., 2008; Lassen et al., 2009; Casado et al., 2013, 2018; Cabrera-Rodriguez et al., 2019; Kafando et al., 2019).

To further investigate the role of viral Env in HIV-1 infection and pathogenesis, in this work, we expanded our previous studies to viral Envs from other sets of viruses isolated from non-clustered LTNP-EC and Viremic LTNP (vLTNP) individuals in comparison with those from groups of chronic progressors patients. Clonal full-length *env* genes derived from viruses of individuals in these distinct clinical groups were analyzed for expression, CD4 dependent-Env-mediated fusion, cell-to-cell viral transfer, and infection efficiency. This analysis permitted the establishment of a relationship between the initial events of the viral replication cycle, mediated by the viral Env characteristics, with the viral load (VL) control and the clinical outcome and pathogenesis of the HIV-1 infection.

## Materials and Methods

### Viral Envelopes

Forty-one viral envelopes (Envs) were obtained from samples of different origins: the HIV HGM BioBank integrated in the Spanish AIDS Research Network (RIS-RETIC, ISCIII) (samples 1–3, 6–8, and 13–19), the Centro Sanitario Sandoval, Hospital Clínico San Carlos (samples 21, 22, 24, 28, 30–33, 36–40, 42–46, and 49–52), the IrsiCaixa Research Foundation (samples 9–12), and from Hospital Xeral de Vigo (samples 26, 27). Samples were obtained in three different phases of the Spanish epidemic from 1993 to 1994, 2004 to 2005, and 2013 to 2014. Samples were processed following current procedures and frozen immediately after their reception. Identification numbers and characteristics are found in Table 1. Although the viral sampling was limited, we tried to select in every individual the most different clones that best represented the viral heterogeneity. The obtention of many clones was difficult because of several factors: first, in LTNPs, both EC and Viremic, the viral and proviral load were low (see Table 1) and thus it was difficult to obtain many clones. In addition, these clones, because of the homogeneity of these populations, were very similar. In contrast, in progressor patients, diversity was higher and it was challenging to obtain many functional clones from each patient, but we sampled two of the more diverse clones. Due to these problems, we obtained only 54 functional clones, from which we selected 41 representing the more diverse ones found in each individual (see Table 1). This selection of clones can be seen, for example, in LTNP EC individual MDM (samples 9–11) infected with two viruses where we selected clones from the two viruses and with different sampling times (samples 9–12). Also, in clones 22 and 24 from individual 64 (viremic LTNP) we studied two samples corresponding to different years. In the selection of the distinct clones from each individual, we also took advantage of a viral dating methodology as previously reported (Bello et al., 2004).

**Table 1:** Epidemiological, clinical, and host characteristics of the viral Envs.

Clinical group	Sub-group	Env code^a^	Patient ID code	Viral Load^g^ (at sampling)	Diagnostic time	Sampling time	Viral dating^b^	HLA-B^i^
LTNP	EC	1	2,057,906-3	<50	1993	2004	1989	4901/5701
		2	3,227,050	<50	1988	2004	1991	0702/5201
		3	3,227,058-3	<50	1992	2004	1991	1402/1402
		6	20,044,616-3	<50	1998	2004	1999	1501/5703
		7^*^	10,246,788	<50	1992	2005	1993	4402/5701
		8		<50	1992	2005	1993	4402/5701
		9	MDM^c^	507	1998	1996	1987	4402/3501
		10	c	<50	1998	2011	1996	4402/3501
		11	c	<50	1998	2005	1996	4402/3501
		12	c	<50	1998	2005	1996	4402/3501
	Viremic	13	4,022,834	3.710	1994	2004	ND	1401/4403
		14	9,684	2.557	1998	2005	1994	1302/4001
		15	2,988,465	2.286	1993	2004	1999	1402/2705
		16	38 17 5	418	1996	2014	1999	2705/5801
		17		418	1996	2014	1999	2705/5801
		18		418	1996	2014	1999	2705/5801
		19		418	1996	2014	1999	2705/5801
		21	30	7.597	1989	1998	2000	1501/3501
		22	64	11.926	1989	1999	1999	4402/4901
		24		11.926	1989	2002	1999	4402/4901
Progressor	Old	26	V10^d^	N.D.^h^	1993	1994	1999	4002/4402
		27		N.D.^h^	1993	1994	1999	4002/4402
		28	V13	N.D.^h^	1992	1994	1990	0702/1402
		30	L10	89.000	----	1993	1993	1501/4901
		31		89.000	----	1993	1993	1501/4901
		32	L 11	42.000	1993	1993	2000	1801/5101
		33		42.000	1993	1993	2000	1801/5101
		36	I14^d^	130.000	1987	1994	2002	0702/3502
		37	d	130.000	1987	1994	2002	0702/3502
		38	I18	170.000	1991	1994	1990	1402/4403
	Modern^e^	39	ESI 17A	156.300	2013	2013	N.A^f^	4201/4402
		40		156.300	2013	2013		4201/4402
		42	ESI 39A	137.700	2012	2014	N.A.	1517/3801
		43		137.700	2012	2014		1517/3801
		44	ESI 41A	129.700	2012	2014	N.A.	3503/5701
		45		129.700	2012	2014		3503/5701
		46	ESI 5A 2	49.107	2004	2007	N.A.	4102/4402
		49	ESI 42 A	11.510	2011	2014	N.A.	1402/4403
		50		11.510	2011	2014		1402/4403
		51	ESI 42 B	41.090	2011	2014	N.A.	0702/1501
		52		41.090	2011	2014		0702/1501

a HIV-1 Env number used in this study and identification codes.

b According to Bello et al. (2004).

c Double infected individual (Casado et al., 2007).

d Individuals with a short antiviral therapy [AZT (zidovudine) and DDI (didanosine) for V10 patient and AZT for I14 patient].

e The Modern Individuals have been infected within 3 years.

f N.A.: not applicable.

g HIV RNA copies/ml.

h N.D.: not done. These patients presented AIDS associated symptoms (CDC A2 stage for V10 patient, and CDC A3 stage for V13 patient) with CD4^+^ T levels of 342 cells/ml (V10 patient) and 76 cells/ml (V13 patient).

i HLA-B diversity is observed between all these individuals.

* Envs isolated from the same patient are indicated by brackets.

### Ethics Statement

Samples were obtained from participants who gave informed consent for genetic analysis studies and they were registered as sample collection in the Spanish National Registry of Biobanks for Biomedical Research with number C.0004030. The consents were approved by the Ethical and Investigation Committees of the “Centro Sanitario Sandoval” (Madrid) and the samples were encoded and de-identified in these Centers. All clinical investigations were conducted according to the principles expressed in the Declaration of Helsinki. The studies were approved by the Comité de Ética de la Investigación y de Bienestar Animal of the Instituto de Salud Carlos III with CEI PI 05_2010-v3 and CEI PI 09-2013 numbers.

### Generation of *env* Gene Expression Plasmids

The *env* genes were amplified at limiting dilution by nested PCR from proviral DNA. The products, designated primary thereafter, were cloned into the pcDNA3.1D/V5-His’s Topo expression vector (Invitrogen) and NL4.3. The R5-tropic BaL.01-*env* (catalog number 11445) glycoprotein plasmid was from the NIH AIDS Research and Reference Reagent Program. Ten viral Envs were derived from 6 LTNP-EC patients, 10 clones from 6 Viremic LTNPs, 10 clones from 6 “Old” individuals (contemporary to LTNPs), and 11 clones from 10 recent “Modern” patients together with NL4.3 and BaL.01 reference clones. Expression plasmids were transformed in DH5α cells, and clones sequenced to check the correct insertion of the *env* gene. Tropism of *env* sequences was determined with the PSSM bioinformatic tool (Web PSSM),^1^ all of them presenting CCR5-tropism (R5-tropism).

### Env Expression and Fusion Assays

The Env expression plasmids were used to transfect HEK-293T cells with X-tremeGENE HP DNA Transfection Reagent (Sigma) in combination with either a Tat expression plasmid pTat for Env expression and fusion assays, or with the *env* defective HIV-1 backbone pSG3 plasmid for viral transfer assays (Curriu et al., 2012; Casado et al., 2018; Cabrera-Rodriguez et al., 2019). As a negative control, HEK-293T cells were transfected only with pTat and as a positive control we use the BaL and NL4.3 Envs. HEK-293T cells were chosen as effector cells since they provide sensitive measures of fusion even when using low fusogenic Env. Twenty-four hours post-transfection, cells were collected, and tested for Env surface expression and also fusion activity.

To test Env expression, 1 × 10^5^ Env/Tat co-transfected HEK-293T cells were incubated with 2G12 and IgGb12 monoclonal antibodies (mAbs; Polymun, Viena, Austria) at 6 μg/ml each for 45 min at RT. After washing the cells, the PE-labeled goat anti-human IgG (Jackson ImmunoResearch Laboratories) was added and incubated in the dark at room temperature for 15 min, as similarly reported (Casado et al., 2018; Cabrera-Rodriguez et al., 2019). Cells were washed, fixed in formaldehyde 1%, acquired in a Celesta flow cytometer (BD FACS Celesta) and analyzed using the Flow-Jo software (Tree Star Inc.) The percentage of Env-positive cells and the Mean Fluorescence Intensity (MFI) of these cells were used to evaluate Env expression.

**Figure 1:**
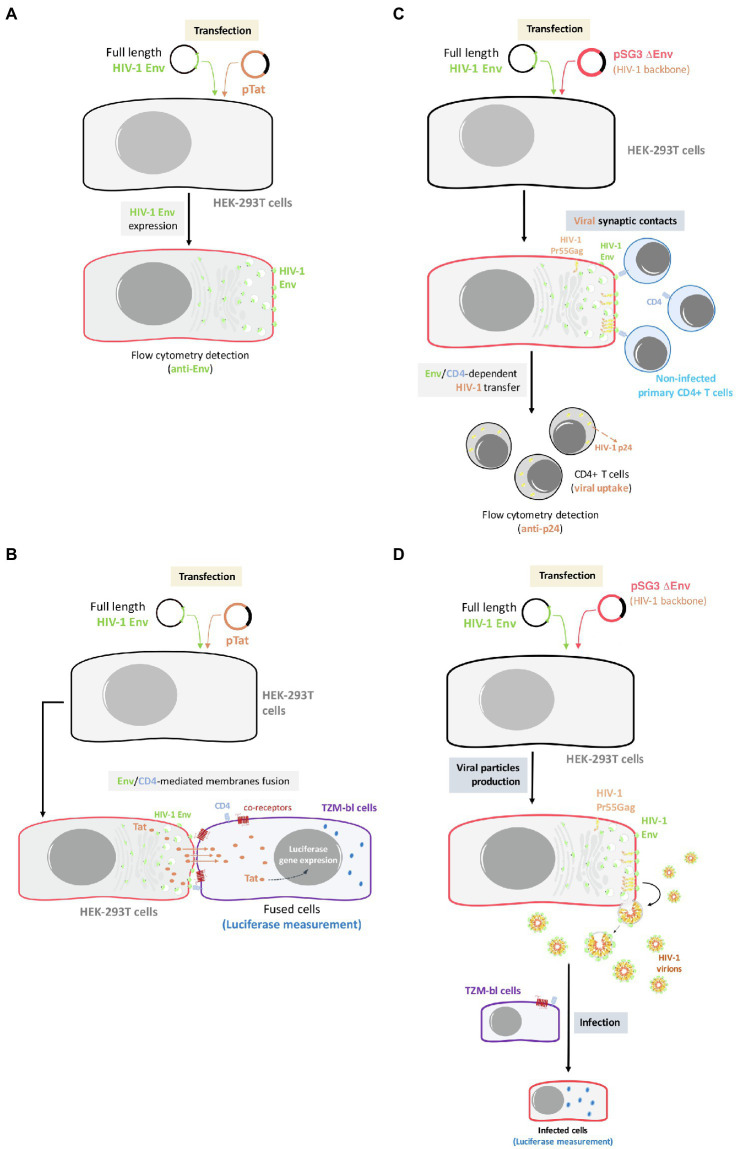
Outline of the experimental model used for the analysis of Env expression, Env-mediated cell-to-cell fusion, viral transfer, and viral infectivity. **(A)** Env expression: HEK-293T cells will be co-transfected with primary or reference full-length viral *env* and a ptat Δ*env* HIV-1 expression plasmid, allowing Env cell-surface expression in a viral production context. Cell-surface Env expression will be then analyzed by flow cytometry using specific anti-Env antibody. **(B)** Env-mediated fusion activity: effector HEK-293T cells producing HIV-1 particles bearing primary or reference Envs will be co-cultured with TZM-bl cells to force synapsis formation and CD4-mediated binding of budding particles to target cells. **(C)** Env-mediated viral transfer: HEK-293T cells producing HIV-1 particles carrying primary or reference Envs will be co-cultured with primary CD4^+^ T cells. Then, HIV-1 transfer will be analyzed by flow cytometry using specific anti-p24 antibody in target CD4^+^ T cells. **(D)** Env-mediated viral infection: TZM-bl cells will be infected with serial dilutions of viral particles obtained from transfected HEK-293T carrying the different primary or reference HIV-1 Envs. After 48 h, infectivity capacity will be analyzed by quantifying luciferase assay in infected TZM-bl cells.

To test fusion activity, 1 × 10^4^ Env/Tat-transfected or control Tat-transfected HEK-293T cells were mixed (ratio 1:1) in 96-well plates with CD4^+^CXCR4^+^CCR5^+^ TZM-bl reporter cells for 6 h at 37°C. Luciferase activity resulting from Tat mediated transactivation was measured (Fluoroskan Accent, Labsystems) using Brite-Lite (PerkinElmer) and normalized to BaL-Env-mediated fusion. The assay provides an indirect but quantitative measure of fusion events (Cunyat et al., 2012). NL4.3 and BaL-Env expression plasmids were used as positive controls for Env staining and as reference value for fusion activity (BaL = 100%), as similarly reported (Curriu et al., 2012; Cabrera-Rodriguez et al., 2019; summarized in the scheme of Figure 1B).

### HIV-1 Transfer/CD4 Binding

To test viral transfer activity, which exclusively depends on the binding of gp120 to the CD4 molecule, Env expression plasmids were co-transfected with the Env-defective pSG3 plasmid in HEK-293T cells, as similarly reported (Curriu et al., 2012; Casado et al., 2018; Cabrera-Rodriguez et al., 2019). One day after transfection, 1 × 10^5^ HEK-293T cells were mixed at a 1:1 ratio in 96-well plates with primary CD4^+^ T lymphocytes freshly isolated from healthy donors by negative selection (CD4^+^ T-Cell Isolation Kit II, human, Miltenyi Biotec). Viral transfer was assessed after 24 h of incubation at 37°C in permeabilized (FIX & PERM Cell Permeabilization kit, Invitrogen Life Technologies) and stained cells with the anti-HIV-1 p24 KC57 mAb (anti-HIV core antigen RD1 labelled, IZASA) for 20 min in the dark at RT. Then, the cells were washed and fixed in formaldehyde 1%, and acquired in a Celesta flow cytometer (BD FACS Celesta), and the content of p24 in gated CD4^+^ T cells and gated HEK-293 T cells was analyzed using the Flow-Jo software (Tree Star Inc.). The percentage of p24+ HEK-293 T cells was used as a control for transfection efficiency and was similar among all experiments, as well as the p24/Pr55Gag level of expression per cell (see Supplementary Figure S1). Since co-receptor binding or fusion activity are not required for viral transfer, the frequency of p24+/CD4^+^ T cells was a direct measure of the amounts of HIV-1 virions bound to or taken up by target cells and reflects the efficacy of Env to bind to cell-surface expressed CD4 molecules (summarized in the scheme of Figure 1C).

### Infectivity Assay

Cloned viral Envs were used to generate pseudoviruses by co-transfection with pSG3 plasmid of HEK-293T cells as indicated above and tested in TZM-bl cells to determine the infectivity capacity. Serial dilutions of the pseudoviruses generated with the different Envs of the different groups of patients were made in a 96-well plate. Then, 1 × 10^5^ TZM-bl cells were added to the pseudoviruses with DEAE dextran hydrochloride (Sigma) at 18 μg/ml. After 48 h of incubation at 37°C, luciferase activity was measured (Fluoroskan Accent, Labsystems) using Brite-Lite (PerkinElmer). Uninfected TZM-bl cells were used as a negative control. The TCID_50_ (Median Tissue Culture Infectious Dose) value was calculated with Montefiori template and normalized with the viral concentrations (summarized in the scheme of Figure 1D).

### Phylogenetic Analysis

The evolutionary history and the HIV-1 subtype B most recent common ancestor (MRCA) nucleotide sequence were inferred by using the Maximum Likelihood method and General Time Reversible model (Nei and Kumar, 2000). Initial trees for the heuristic search were obtained automatically by applying Neighbor-Join and BioNJ algorithms to a matrix of pairwise distances estimated using the Maximum Composite Likelihood (MCL) approach, and then selecting the topology with superior log likelihood value. A discrete Gamma distribution was used to model evolutionary rate differences among sites [five categories (+G, parameter = 0, 4, 590)]. The rate variation model allowed for some sites to be evolutionarily invariable [(+I), 14, 43% sites]. This analysis involved 142 nucleotide sequences. Codon positions included were 1st + 2nd + 3rd + Noncoding. All positions with less than 95% site coverage were eliminated, i.e., fewer than 5% alignment gaps, missing data, and ambiguous bases were allowed at any position (partial deletion option). There were a total of 2,454 positions in the final dataset. Evolutionary analyses were conducted in MEGA X (Kumar et al., 2018). Evolutionary divergence from subtype B MRCA was estimated as the number of base substitutions per site between each nucleotide sequence analyzed and HIV-1 subtype B MRCA sequence using the Maximum Composite Likelihood model.

Nucleotide sequences have been deposited in GeneBank under the following numbers: KC595156, KC595162, KC595225, KC595227, KC 595189, MH605987, MH605986, KC595190, MH605988, MH605992, MH605991, MH605970, MH605971, KC595223, KC595222, MH605972, MH605975, MH605976, MH605978, MH605973, MH605979, MH605980, MH605981, MH605982, MH605983, MH605984, MK394184, and MK394185.

### Statistical Analysis

Data and statistical analyses were performed using GraphPad Prism, version 6.07 (GraphPad Software). Significance when comparing groups was determined with a nonparametric Kruskal–Wallis or by nonparametric Dunn’s test for multiple comparisons. A nonparametric Spearman test was used to calculate correlations. A heatmap was graphed representing the normalized experimental measures. These variables and Env clones were grouped by hierarchical cluster analysis using Ward’s minimum variance method.

### Data Availability Statement

All “accession numbers” and “data” of this work are available.

## Results

### Phylogentic Analysis of the Viral Envelope Sequences

For the investigation of the potential role of the HIV-1 Env in virological control and pathogenesis, we studied the phenotypic characteristics of 41 Envs from 24 individuals without antiviral therapy and different VLs (Table 1). We analyzed 10 Envs from six LTNP-EC individuals with undetectable VL, no clinical progression and infected in the late 80s and 90s; 10 viral clones from six Viremic LTNPs (vLTNPs) with VL < 10,000 viral copies/ml and infected in the 90s. The functional characteristics of these viral Envs were studied by following the experimental strategy presented in Figure 1.

**Figure 2:**
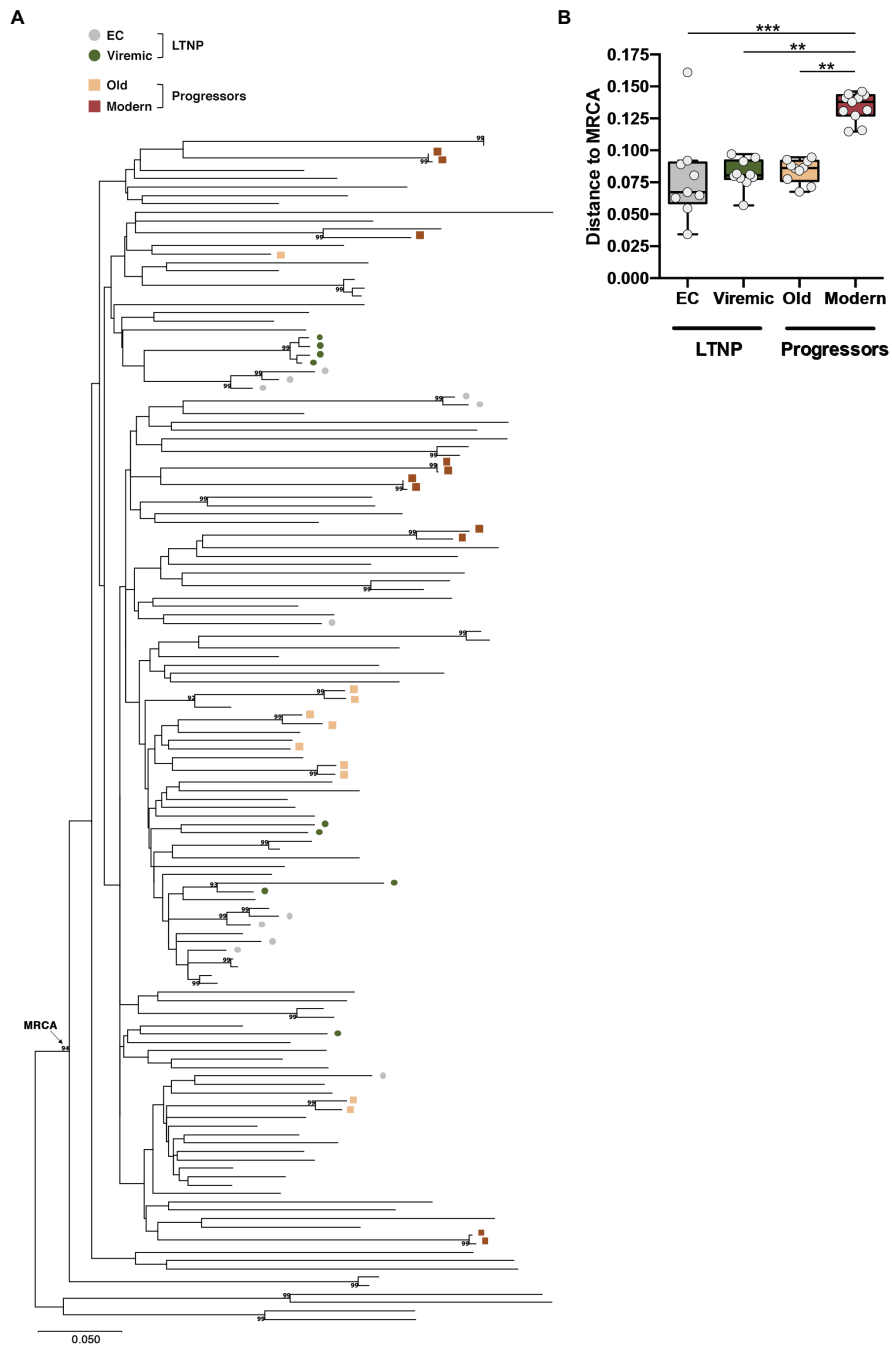
Phylogenetic analysis of the viral envelope sequences relate to the most recent common ancestor (MRCA). **(A)** Phylogenetic tree and subtype B MRCA were inferred with complete viral env nucleotide sequences by using the Maximum Likelihood method and General Time Reversible model using MEGA X software X (Kumar et al., 2018). The percentage of trees in which the associated taxa clustered together with values over 90% is shown next to the branches. The tree is drawn to scale, with branch lengths measured in the number of substitutions per site. The phylogenetic analysis of the studied viral sequence (the env gene for LTNP-ECs and vLTNPs, and Old and Modern progressors) did not reveal relationships between the different groups and no clustering except for those sequences obtained from the same individual. **(B)** Box-plot representation of the genetic distances of the viral env sequences to the MRCA of LTNP viruses (EC and Viremic) compared with the MRCA-genetic distances of viruses obtained from HIV-1+ progressor individuals (Old and Modern); values of *p* (Kruskal–Wallis test) for comparison between all groups are shown. When indicated, *p* values are **p<0.05 and ***p<0.001.

To ascertain that the characteristics of the Envs from the LTNPs were not due to the sampling time, we compared them with 10 Envs obtained from six HIV-1 individuals also infected in the same period (90s), designated Old, but with high VL > 10^5^ viral copies/mL and chronic infections. Finally, we studied 11 viral clones from six chronic individuals infected between 2013 and 2014 with VL > 10^4^ viral copies/ml. The main characteristics of the participants are summarized in Table 1. The phylogenetic analysis of the viral sequence in the *env* gene did not reveal associations between the different groups and not any clustering except for those sequences obtained from the same individual (Figure 2A). Envs from LTNP-ECs and one vLTNPs grouped in short branches, as a consequence of the viral and evolutionary control, whereas long branch length was observed in the sequences from non-controller patients (Old and Modern), because of the higher replication and viral evolution in these individuals (Figure 2A). The estimation of the viral dating of the sequences from the different groups (Table 1) and the distance to the MRCA supported the clinical classification of the studied individuals and gave information of the individual viral evolution (Figure 2B, *box–plot*). The sequences from the LTNP-ECs showed a viral dating of 1992,2, those from the viremic LTNPs was 1998,2, and those from the Old individuals 1995,6. Thus, the viral dating and the distance to MRCA showed the limited viral evolution in LTNPs particularly in the ECs, in contrast with the wider evolution and more recent isolation in the progressor individuals (Old and Modern).

### Analysis of Cell-to-Cell Membrane Fusion and Viral Transfer Capacity of Viral Envelopes

**Figure 3:**
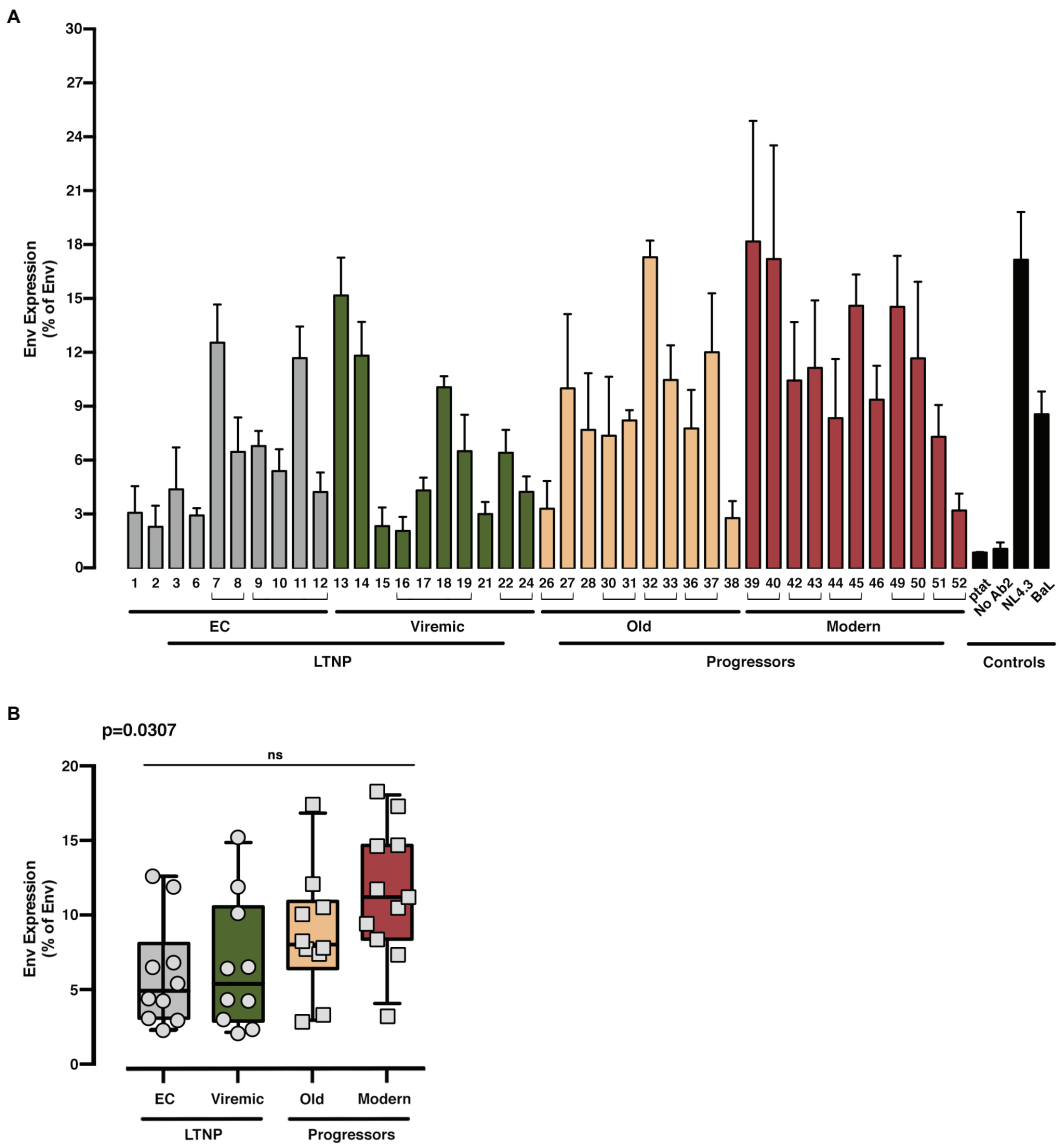
Analysis of the expression of the different HIV-1-Env glycoproteins from LTNP-EC, Viremic LTNP, and control progressors patients. Flow cytometry analysis of the cell-surface expression level of the assayed HIV-1 Envs in HEK-293T cells from LTNP-EC (*gray bars*), vLTNP (*green bars*), Old (*orange bars*), and Modern individuals (*red bars*) or reference HIV-1 viral strains (ptat, No Ab2, NL4.3, and BaL, *black bars*). Env protein expression for each patient **(A)** and Env protein expression in each group of patients comparing mean values between each group (Kruskal–Wallis, Dunn’s Multiple Comparisons Test; **B**) are shown. Envs isolated from the same patient are associated with brackets in **(A)**; value of *p* (Kruskal–Wallis test) for comparison between all groups is shown, *top left*. No significant (ns) differences were identified after multiple comparison analysis. Values are mean ± SEM of three independent experiments (*n* = 3). When indicated, comparative *p* values between groups are ***p<0.001 and ****p<0.0001.

With these Env, we first analyzed the potential differences in the expression between the Env clones from the clinical groups, by measuring their cell-surface expression levels in HEK-293T cells (Figure 1A *shows study scheme*, and Figure 3). Although we observed a higher median expression in Env viral clones derived from patients that do not control viremia (i.e., Old and Modern patients) compared to LTNPs (EC and Viremic), this increase did not reach statistical significance among groups (Figure 3). Thus, the expression capability of the viral Envs appears to not contribute to the differences in VL and pathogenesis between groups.

A key process for HIV Env-mediated infection is the interaction of the Env complex with the CD4 receptor. When this interaction is functionally efficient, viral transfers through synaptic contacts or fusion pore formation are triggered during cell-to-cell or virus-to-cell contacts, respectively (Blanco et al., 2004; Valenzuela-Fernandez et al., 2005; Barrero-Villar et al., 2009; Casado et al., 2018; Cabrera-Rodriguez et al., 2019).

**Figure 4:**
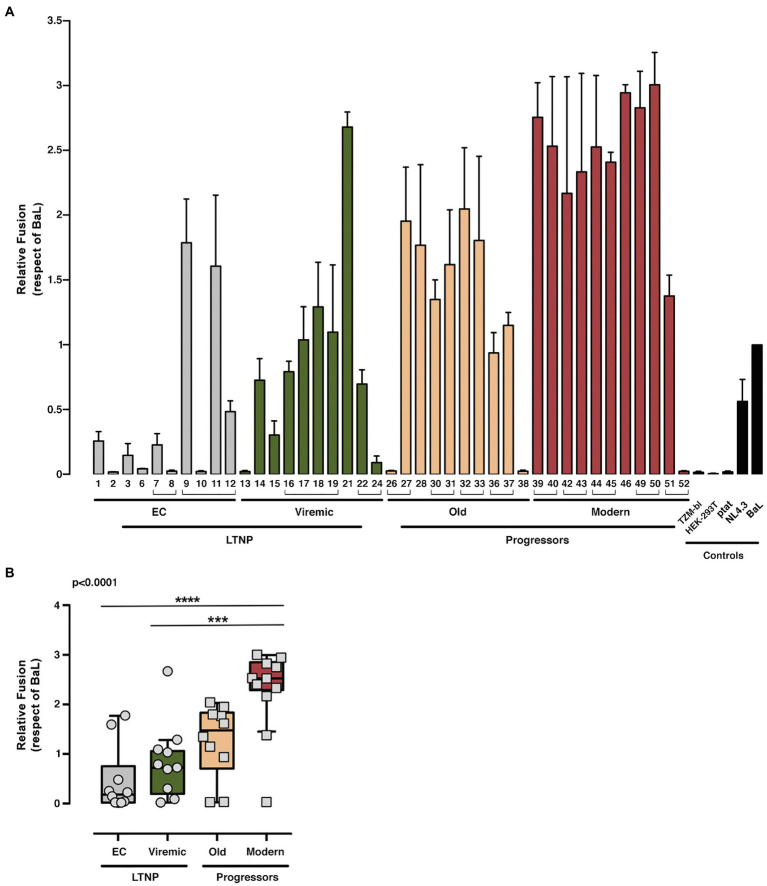
Analysis of membranes fusion-phenotypic features of HIV-1 Envs isolated from LTNP-EC, viremic LTNP, and P individuals. Analysis of the ability to induce cell-to-cell fusion of HIV-1 Env proteins obtained from LTNP-EC (*gray bars*), vLTNP (*green bars*), Old (*orange bars*), and Modern individuals (*red bars*) or reference HIV-1 viral strains (ptat, NL4.3, and BaL, *black bars*). **(A)** Env fusogenic activity for each patient in each group is shown. Envs isolated from the same patient are associated with brackets. **(B)** Relative fusion activity of the full Env collection compared to the BaL control established at 100% and grouped in the different groups of patients is shown. Values are mean ± SEM of three independent experiments (*n* = 3). Statistical analysis was performed using Kruskal–Wallis, Dunn’s Multiple Comparisons Test; value of *p* for comparison between all groups is shown, *top left*. When indicated, comparative *p* values between groups are ***p<0.001 and ****p<0.0001.

**Figure 5:**
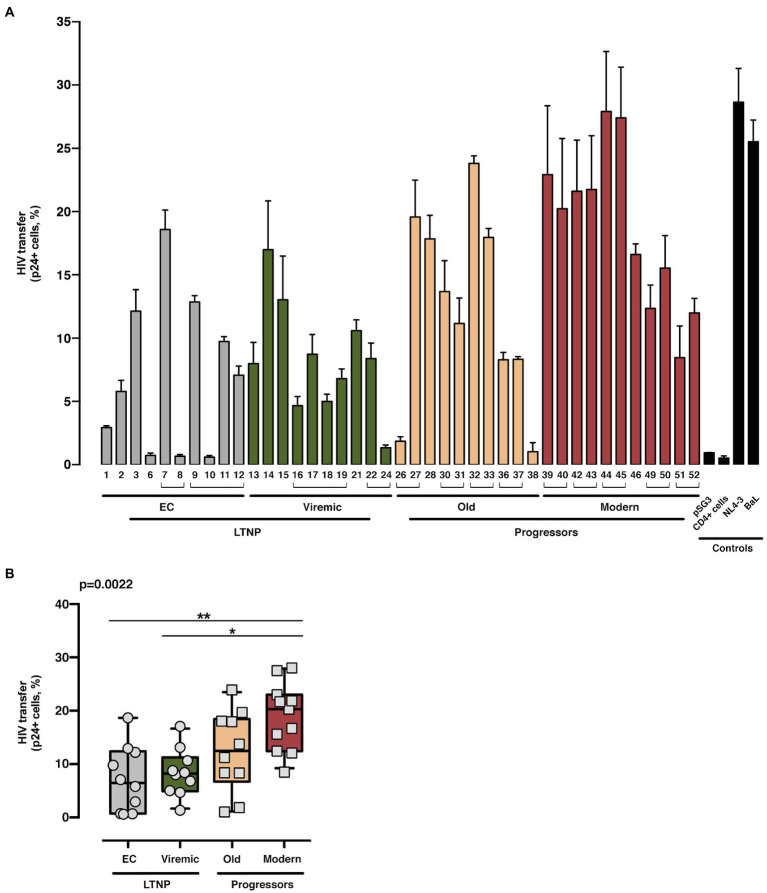
Analysis of HIV-1 Env-mediated cell-to-cell viral transfer. Analysis of the ability to induce cell-to-cell virus transfer of HIV-1 Env proteins obtained from LTNP-EC (*gray bars*), vLTNP (*green bars*), Old Patients (*orange bars*), recent patients (Moderns; *red bars*), or reference HIV-1 viral strains (pSG3, CD4^+^ cells, NL4.3, and BaL, *black bars*). Analysis of HIV-1 Env-mediated cell-to-cell viral transfer for each patient **(A)** and in each group where values of *p* compare medians between groups using a nonparametric Kruskal–Wallis Test (Kruskal–Wallis, Dunn’s Multiple Comparisons Test; **B**) are shown. Envs isolated from the same patient are associated with brackets in **(A)**; value of *p* for comparison between all groups is shown, *top left*. *p* values are *p<0.01 and **p<0.05. Values are mean ± SEM of two independent experiments (*n* = 2).

We studied the viral Env/CD4 interaction and the efficiency of subsequent functions, measuring the membrane fusion capacity of the Envs by analyzing Tat-mediated activation of LTR (Figure 1B *shows study scheme*) in co-cultures between Env-expressing HEK-293T and HIV-permissive target TZM-bl cells (Figure 4). To fully characterize our experimental models, we used the Envs from reference HIV-1_BaL_ (CCR5-tropic) and HIV-1_NL4.3_ (CXCR4-tropic) viruses (Figures 4, 5). This fusion assay yielded lower fusion values for Envs of viruses from LTNP-ECs and from vLTNPs than for Old and Modern progressors, and attaining statistical significance between LTNPs (EC and Viremic) and Modern Env glycoproteins (Figure 4B).

Next, we assayed the CD4-dependent cell-to-cell virus transfer capacity of the viral Envs (Figure 5). This experiment was performed co-culturing Env-expressing HEK-293T cells with unstimulated primary CD4^+^ T lymphocytes as target cells (Figure 1C *shows study scheme*, and Materials and Methods). In this assay, we forced the formation of virological synapses, which exclusively relies on binding of Env to CD4, between virus-effector HEK-293T cells expressing the different Envs together with the structural HIV-1 Gag polyprotein (p24 flow cytometry analysis in Supplementary Figure S1), and fresh primary CD4^+^ T cells from healthy donors (Figure 1C *shows study scheme*). The Envs from the LTNPs (EC and Viremic) individuals displayed a lower ability to transfer viral particles to primary CD4^+^ T lymphocytes than Envs from Old individuals and significantly lower than from Modern participants (*p* < 0.0022 between all groups; Figure 5). These data suggest that the Envs from LTNP-EC viruses had an impaired binding to the cell-surface CD4 receptor and that this impairment was progressively overcome in the Envs from individuals from the other groups with less control of viral replication and higher VL.

Thus, the phenotypic characterization of the Envs of viruses from subjects with distinct progression rates confirmed that LTNP-ECs and vLTNPs presented viruses with impaired Env CD4-associated functions and a significant lower fusogenic and transfer capacity, in comparison with viruses from the viremic groups. These lower characteristics (Figures 4, 5) were also linked with the low VL detected in these subjects (Table 1). Furthermore, we observed a functional improvement in the viral Envs from the LTNP-EC and vLTNP individuals to those of chronic Modern glycoproteins: these data support that the deficient Env fusion and transfer capacities observed in the Envs of viruses from LTNP-EC and vLTNP phenotypes have been enhanced in the viruses from individuals with progressive infection, particularly in those of the Modern group.

### Infectivity of Recombinant Viruses With the Analyzed Envelopes

**Figure 6:**
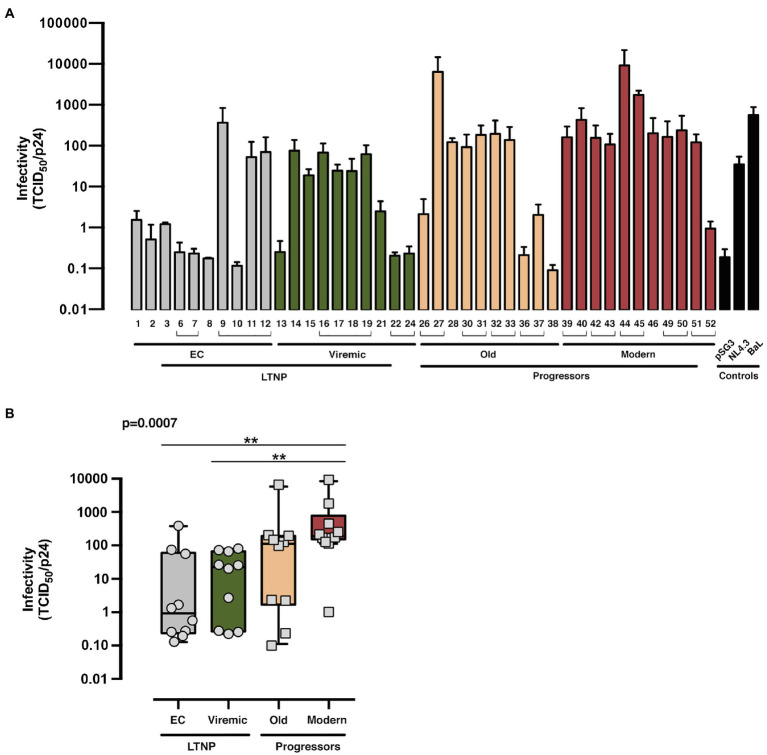
Viral infectivity of the viral Envs. Analysis of the infecivity (TCID_50_ value normalized by viral p24 input) of the different of HIV-1 Env proteins obtained from LTNP-EC (*gray bars*), vLTNP (*green bars*), Old (*orange bars*), and Moderns (*red bars*) patients or reference HIV-1 viral strains (pSG3, NL4.3, and BaL, *black bars*). Analysis of Env infectivity for each patient **(A)** and in each group where values of *p* compare medians between groups using a nonparametric Kruskal–Wallis, Dunn’s Multiple Comparisons Test **(B)** are shown. Envs isolated from the same patient are associated with brackets in **(A)**; p value for comparison between all groups is shown, *top left*. When indicated, comparative *p* value is **p<0.05. Values are mean ± SEM of three independent experiments (*n* = 3).

For the exploration of the potential consequences of these Env properties in virus biology, we estimated the infectivity of recombinant viruses bearing the Env from the different HIV+ phenotypic groups in TZM-bl cells (Figure 1D *shows study scheme*, Figure 6, and Supplementary Figure S1, *flow cytometry analysis of p24/Pr55Gag expression*). Viral Envs from the LTNP-EC group showed the lowest infectivity values, whereas the Modern Envs produced the higher titers. The viruses from vLTNPs displayed higher titers than LTNP-ECs but lower than those from Old individuals. Recombinant viruses from individuals with high VL and progressive infection (Old and Modern) have higher infectivity rates than those with viral control (EC and Viremic). These results demonstrated why the viral properties analyzed (binding, fusion, and transfer) have a significant impact in viral infectivity with an important effect in the biology of HIV-1 and viral pathogenesis.

### HIV-1 Envelope Amino Acid Sequences Analysis

For the search of potential mechanisms involved in the changes of the characteristics among the different Envs sets, we analyzed the Env amino-acid (aa) sequences that could be associated with the distinct clinical phenotypes. As presented in Figure 2A, we performed a phylogenetic reconstruction from *env* nucleotide sequences together with other nucleotide sequences obtained from HIV-1 Spanish individuals. All nucleotide sequences analyzed correspond to HIV-1 subtype B with a CCR5 phenotype. This analysis did not reveal phylogenetic relationships between the different groups analyzed and no clustering except for those nucleotide sequences obtained from the same individual (Figure 2).

**Figure 7:**
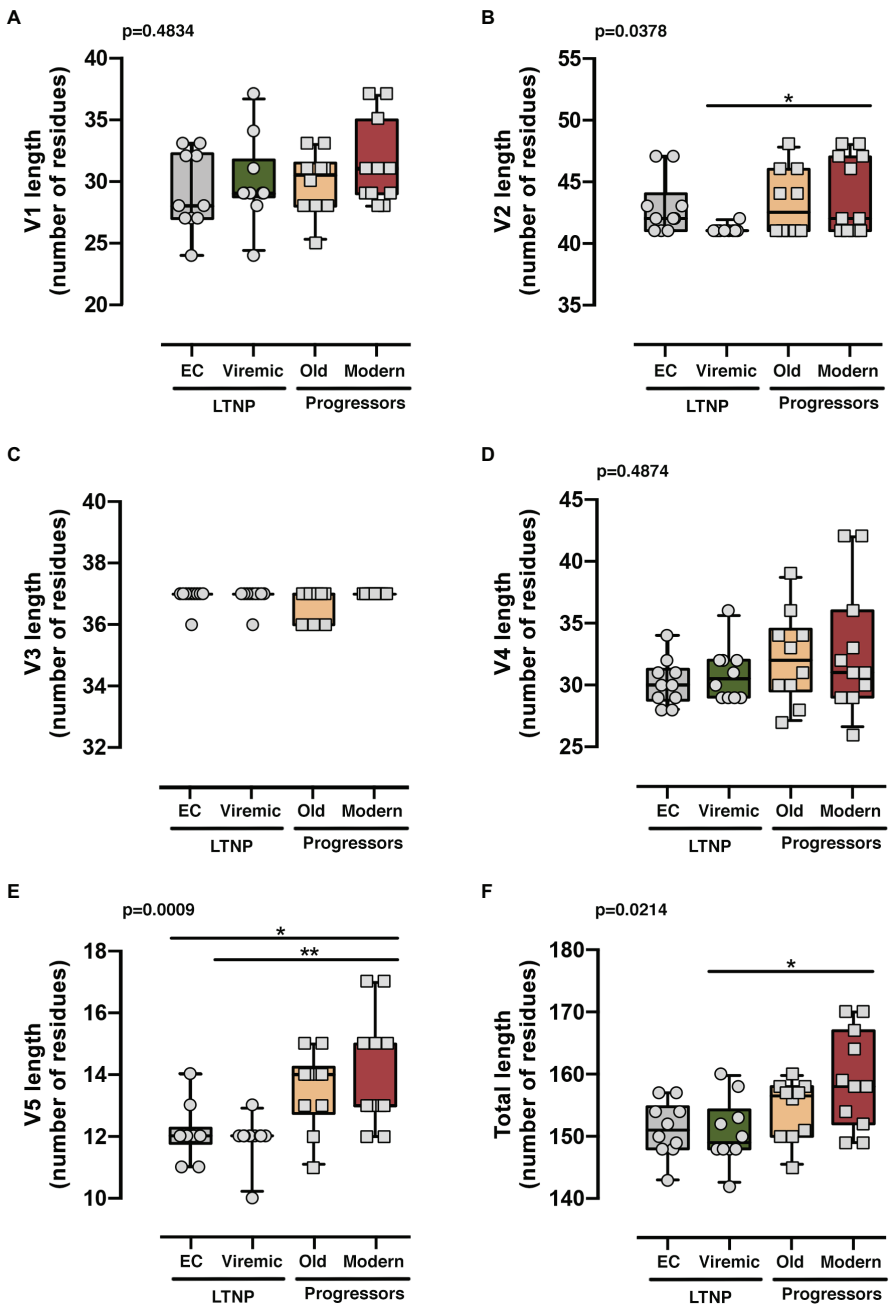
Analysis of the length of the loops of the Envs from the different groups. Analysis of the length of each variable loops V1 **(A)**, V2 **(B)**, V3 **(C)**, V4 **(D)**, V5 **(E)**, and all variable loops together **(F)**. The results were grouped [LTNP-ECs: *gray bar*, vLTNPs: *green bar*, Old patients: *orange bar*, and recent patients (Moderns): *red bar*] and compared using a nonparametric Kruskal–Wallis, Dunn’s Multiple Comparisons Test; p value for comparison between all groups is shown, *top left*. When indicated, comparative *p* values between groups are *p<0.01 and **p<0.05. Lines are mean, boxes 25th and 75th percentiles, and bars minimal and maximal values, considering the characteristics (i.e., loop length) of all Envs analyzed from the different groups.

We then carried out a comprehensive study of the protein sequences focusing in the variable loops and their associated potential N-linked glycosylation sites (PNGs) in the gp120 subunit of the Env. In general, as previously reported, there is a trend in the HIV-1 viral Env to gain length and glycosylation sites along the epidemic (Sagar et al., 2006; Curlin et al., 2010; Yuan et al., 2013). This increasing trend is also found in our work where viruses from the LTNPs (EC, Viremic) and Old Envs isolated in the 90s showed shorter lengths than those of the Modern group obtained in 2013–2014 (Table 2). The V3 loop was the most conserved and constant region in length and glycosylation sites (Table 2 and Figure 7), while the other loops showed length increases predominantly in the V2 and V5 loops that were reproduced in the total length (Table 2 and Figure 7). The only statistical differences were noticed between the total length in the LTNPs (EC and Viremic) vs. Modern Envs in V5 and in V2 between viremic LTNP and Modern (Figure 7).

**Table 2:** Molecular characteristics of HIV-1 Envs: sequence length and N potential glycosylation sites (PNGs) in the variable loops (Vn) of the gp120 subunit.

Clinical group	Subgroup	Env code	V1/G^a^	V2/G	V3/G	V4/G	V5/G	ΣVn/G^b^	Mean/G^c^	Gp160^d^	Mean^e^
LTNP	EC	1	28/4	43/2	37/2	28/4	12/1	148/13		848	
		2	33/5	41/2	37/2	31/4	12/1	154/14		853	
		3	33/5	41/2	37/2	31/3	12/2	154/14		853	
		6	28/3	41/2	37/2	34/4	12/1	152/12		852	
		7^*^	32/5	47/2	37/2	30/4	11/2	157/15	151.1/14.4	859	851.8
		8	32/5	47/2	37/2	30/4	11/2	157/15		859	
		9	24/4	43/2	36/2	28/4	12/1	143/14		843	
		10	27/4	42/2	37/2	29/5	14/2	149/16		851	
		11	27/5	42/3	37/2	29/5	13/2	148/17		850	
		12	27/4	42/3	37/2	32/5	12/1	150/14		850	
	Viremic	13	31/5	41/2	37/2	31/4	13/1	153/14		854	
		14	29/4	42/2	37/2	32/5	12/2	152/15		852	
		15	34/5	41/2	37/2	36/5	12/1	160/15		860	
		16	29/5	41/2	37/1	29/5	12/1	148/14		849	
		17	29/5	41/2	37/2	29/5	12/1	148/16	150,3/14.1	849	851.5
		18	29/4	41/2	37/2	29/5	12/1	148/15		849	
		19	29/4	41/2	37/2	29/5	12/1	148/14		849	
		21	24/3	41/2	37/1	30/5	10/0	142/11		842	
		22	28/4	41/2	37/2	32/5	12/1	150/14		850	
		24	37/7	41/2	36/2	32/5	12/1	158/15		861	
Progressor	Old	26	31/4	41/3	37/2	39/7	14/2	160/18		862	
		27	31/5	48/3	37/2	28/5	14/2	158/16		858	
		28	25/5	41/2	36/2	33/5	12/2	145/15		848	
		30	33/4	41/2	37/2	27/4	11/1	150/14		852	
		31	33/5	41/2	37/2	36/5	13/2	158/16		860	
		32	28/5	44/2	36/2	30/5	15/2	151/15	153,8/15.2	853	855.8
		33	31/4	44/2	36/2	30/5	15/2	156/14		856	
		36	28/4	46/1	37/2	34/5	14/2	157/15		859	
		37	28/4	46/2	37/2	34/5	14/2	157/16		859	
		38	30/4	41/3	37/1	31/4	13/2	150/13		851	
	Modern^e^	39	31/4	41/2	37/2	29/4	12/2	149/14		849	
		40	31/4	41/2	37	29/4	17/2	154/14		849	
		42	29/4	48/2	37	36/6	17/2	167/13		878	
		43	29/4	48/3	37	30/4	15/2	159/15		872	
		44	28/4	47/3	37	31/4	15/2	158/15	158.1/14.7	859	862.0
		45	28/4	47/2	37	31/4	15/2	158/14		859	
		46	35/4	46/3	37	33/5	13/2	164/15		865	
		49	37/6	41/2	37	42/7	13/1	170/18		871	
		50	37/6	41/2	37	42/7	13/1	170/18		871	
		51	31/4	42/2	37	26/3	13/1	149/12		853	
		52	29/4	42/2	37	32/6	12/1	152/15		856	

a Length in amino acid (aa) and potential glycosylation sites (PNGs) of the Env-gp120 variable regions (Vn; from V1 to V5) expressed as Vn/G ratio.

b ΣVn/G indicates the sum of the aa lengths of the Vn (*n*; from 1 to 5) and the potential G sites.

c Mean/G indicates the mean length and PNG value for each group of Envs.

d Gp160 shows the total length in aa of each Env including the gp41 subunit and the gp120 subunit.

e Mean gp160 length in aa for each group of Envs.

* Envs isolated from the same patient are indicated by brackets.

Regarding the PNGS, most of the 24 relevant previously described sites (Sterjovski et al., 2007; Go et al., 2008; Gnanakaran et al., 2011; Wang et al., 2020) were present in these sets of viral glycoproteins. However, major differences were observed in the aa extension of the loops with a progressive acquisition of more PNGS in the Modern Envs (Table 2). Glycan at N289 site was more present in LTNP-ECs, vLTNPs, and Old viruses but is not present in Modern ones. Position N362 which is N proximal to the CD4 binding “DPE” motif (positions 368-370HXB2 sequence) was conserved in LTNP-EC, Viremic, and Old but was only present in two of the Modern Envs. It is interesting to highlight that changes also occurred in the viral transmembrane gp41 protein in glycan N816 that was dominant in LTNPs but not in chronic individuals (Old and Modern).

### Correlation Between Viral Characteristics of the Envelopes

**Figure 8:**
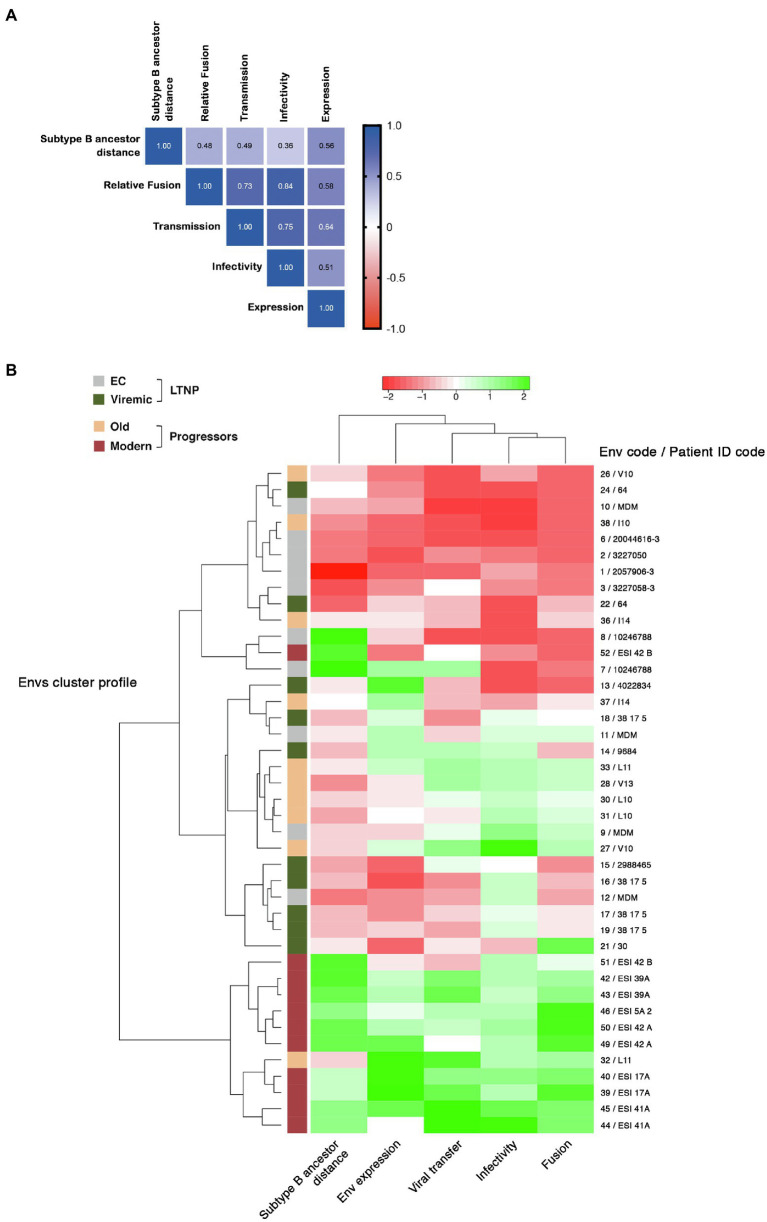
Correlation of the expression, fusion, transfer viral infectivity and genetic distance and clustering of Env characteristics. **(A)** Correlation between genetic distance to subtype B ancestor of Envs of all different groups and Env expression, relative fusion, HIV Transfer, and Infectivity (Spearman correlation values are indicated in the matrix representation). **(B)** Env from LTNP-ECs (*gray*), vLTNPs (*green*), Old patients (*orange*), and Modern patients (*red*) were analyzed according to the indicated parameters. Two main clusters corresponding to EC and modern progressors were identified with opposite functional characteristics.

A significant positive correlation was observed among the different functional parameters analyzed for each Env: expression, cell-to-cell transfer data, which is directly mediated by Env/CD4 binding, infectivity, and fusogenicity (Figure 8A and Supplementary Figure S2, *representing standard correlation graphs*). Furthermore, there was an unsupervised clustering of Env clones according to functional characteristics, clearly grouped Envs from subjects with virological control (EC), and Envs from the modern non-controlling individuals on opposite branches. Therefore, HIV-1 Envs displaying poor viral functions, because of the poor binding of the viral Env to the CD4, correlated with viremic control and non-progressor clinical phenotypes. In contrast, functional Envs are associated with the lack of viremic control and the progressor clinical phenotypes. These statistical correlations support an association between Env-viral properties that directly condition HIV-1 infection, and the viral phenotype which correlate with the clinical phenotype of the HIV+ individuals studied.

It is interesting to mention that the trend in Env length increase follows the same pattern that the functional growth of the Env shown in the distinct viral characteristics (see Figures 4–7). Consistently, we observed a good correlation between the genetic distance to the subtype B MRCA obtained by phylogenetic reconstruction and the functionality of viral Env proteins analyzed (Figures 8A,B and Supplementary Figure S3, *representing standard correlation graphs*). In general, the lower evolutionary sequences (less genetic distance to subtype B MRCA) are those with lower functionality (LTNP-ECs) and the higher evolutionary sequences are those with higher functionality (Moderns). In summary, the viral Envs with the most efficient characteristics are found within the Envs of the Modern group that also show the longer gp160 proteins, with more glycosylated sites and higher distance to the subtype B MRCA.

## Discussion

HIV-1 infected individuals display a wide spectrum of clinical progression rates. The causes of this dispersion are multiple and associated with the operation of numerous combinations of host genetic, immunological, and viral factors. In this work, we studied the potential contribution of viral Env glycoprotein characteristics to the clinical outcome of HIV-1 infection in HIV+ individuals with different clinical status.

The different groups of patients were defined by their clinical characteristics, distinct VLs, because several studies have described a clear correlation between patients’ VL and the likelihood of virus transmission, disease progression, and pathogenesis (Coffin, 1995; Mellors et al., 1995, 1996; Quinn et al., 2000; Fideli et al., 2001; Pilcher et al., 2007; Hecht et al., 2010; Yue et al., 2013). The groups displayed very distinct viral loads and infection times (Table 1) and were representatives of individuals with different clinical characteristics (Casado et al., 2010). The groups exhibited, however, a limited number of Env clones because it is very laborious and difficult to perform the different phenotypic analysis in multiple samples.

The replication of HIV-1 is inevitably associated with the generation of genetic variability that is the basis for viral evolution and the emergence of new variants. The quantification of viral evolution led to the proposal of the existence of a relaxed molecular clock (Casado et al., 2000; Bello et al., 2004) in the Spanish epidemic as previously shown in other countries (Leitner and Albert, 1999), and permitted the estimation of a potential viral dating of a sequence (Casado et al., 2000; Bello et al., 2004). This viral dating also corresponds to the genetic distance to the MRCA.

Although viral control in HIV-1 individuals has been linked to the host-immune responses (Deeks and Walker, 2007; Balasubramaniam et al., 2019), other researchers and our group have established a direct connection between deficiencies in HIV-1 Env-associated functions and long-term viremia control in LTNP-ECs (Lassen et al., 2009; Casado et al., 2013, 2018). The Envs from these LTNP-EC individuals were ineffective in the CD4 binding and in the subsequent functions: viral signaling, fusion, and cell entry. These Env characteristics ensued in low replication and transmissibility of the virus (Valenzuela-Fernandez et al., 2005; Barrero-Villar et al., 2009; Casado et al., 2018; Cabrera-Rodriguez et al., 2019). All these data strongly support the role of the viral Env in the LTNP-EC phenotype and viral pathogenesis.

In the present work, we extended these observations to Envs from non-progressor subjects which are not associated with a cluster of infection, in comparison to different sets of progressor chronic individuals. The Envs characteristics from LTNP individuals (EC and Viremic) were compared with those of individuals with progressive infection (Old and Modern). We investigated the defects in the association of Envs with the CD4, membrane fusion impairment, the cell-to-cell virus transfer, and viral infection capacities. Viral Envs from LTNPs showed the lower binding capacity to the CD4 receptor and this initial inefficient Env/CD4 interaction led to a deficiency in membrane fusion and virus cell-to-cell transfer capabilities, as we also reported for a cluster of LTNP-ECs’ Envs (Casado et al., 2018).

The properties of the Env from LTNPs were not due to the ancestral origin of the LTNPs viruses infected in the late 80s and 90s, because the characteristics of the Old viruses which were contemporary to the LTNPs did not show these limited functional characteristics. On the contrary, Envs from progressors (Old and Modern) presented efficient CD4-mediated viral functionality that triggered an effective membrane fusion and viral transfer. Thus, we disclosed that there is a clear correlation between the level of viral fusion, the transfer capacity of the viral Env, and viral infectivity. The observed differences between the characteristics of the Envs from these groups could not be associated with viral tropism, because all the *env* nucleotide sequences from the studied viruses showed an R5-tropism (Web PSSM; see Footnote 1).

The lack of viral evolution in LTNPs can be clearly seen in the phylogenetic tree (Figure 2), in the viral dating (Table 1), and the distance to the MRCA. These observations and previous identification of genetically related viruses in different LTNP-EC individuals (Casado et al., 2018) support that the viruses in LTNPs are very close to the T/F viruses. In this regard, the inability of the viral Envs of these patients to efficiently bind to CD4 and fuse is related to the deficient viral transmission and infection, which correlates with a very poor viral evolution and diversity.

The immune response has recently been proposed to be related to the control of viral evolution by potentially removing functional evolved viruses from ECs, but these viruses would resist immune-mediated elimination through chromosomal integration into heterochromatin locations conferring deep latency and protecting against immune targeting (Lian et al., 2021). The authors argued that these immune events are not fundamentally different between individuals under antiretroviral therapy (ART) and ECs. Furthermore, it is clear that the poorest viral functions associated with the Envs of the LTNPs and the low variability will help the immune system to control the infection. In this matter, as presented in Table 1, the patients of this study present variability at the level of the B alleles of the human leukocyte antigen (HLA) class I molecule. Likewise, certain HLA-B alleles, such as HLA-B*27, HLA-B*57, and HLA-B*14, are prevalent among ECs and have been associated with enhanced virological control among these individuals (Almeida et al., 2007; Fellay et al., 2007; Pereyra et al., 2010). Therefore, the HLA-B variability observed in these LTNP HIV+ individuals could indicate that the deficient Env functions could help to maintain the virological control in these individuals.

In this context, viral Envs from LTNPs exhibited non-functional characteristics (Figures 4–7) in comparison with those from viruses of the progressive infection groups, supporting that the properties of the Envs directly condition HIV-1 infection. Of note, poor viral functions correlate with viral control and low clinical progression rate in HIV+ LTNP individuals, whereas functional Envs are from viruses linked with HIV+ patients lacking viral control and presenting clinical progression.

In spite of the limited sampling, because of the difficult and laborious characterization of the viral phenotypes, we observed statistically significant differences between the characteristics of the Envs of viruses from LTNP-ECs and the Moderns. Also, if we consider the Env characteristics from all clinical groups, there is a consistent and recurrent tendency to gain functionality in the viral Envs from the LTNP individuals (LTNP-ECs and vLTNPs), to those of the progressive groups (Old and Modern).

Remarkably, the increase in Env functionality also correlated with longer and more glycosylated proteins (Table 2). The aa length and PNGs’ profile of the Envs from the individuals of the distinct clinical groups showed that the studied Envs tend to increase length and glycosylation over the course of the epidemic as previously described (see Sagar et al., 2006; Curlin et al., 2010). We observed that Env changes accumulated essentially in the V1, V2, V4, and V5 loops, as previously shown in works relating the role of V1 and V4 loops in the CD4 binding and neutralization (Rong et al., 2007; Castro et al., 2008; Moore et al., 2008; van Gils et al., 2011) and viral cell-to-cell transfer capacity (Yuan et al., 2013; Davenport et al., 2016; Wang et al., 2019). Regarding specific changes detected in our study, the loss of the N362 PNGs [position in the HXB2 isolate; group M, subtype B (HIV-1 M:B_HXB2R: NCBI:txid11706)] which was prevalent in the EC, Viremic, and Old but not in the Modern Envs groups could be associated with the gain of functionality in the Envs. However, the opposite effect with more efficient fusion and transfer capacity was found in Australian viruses with the N362 glycosylation site (Sterjovski et al., 2007). The potential role of the other changes in PNGs detected in our study needs to be further investigated. Besides these important changes, it is clear that point mutations could have a significant impact in the viral characteristics and HIV pathogenesis (Coffin and Swanstrom, 2013; Mishra et al., 2019). Thus, the contribution of the individual mutations deserves further studies but it is now out of the scope of the present work.

In contrast with the more significant changes detected in the V2 and V5 loops, it is important to point to the stability in length and glycosylation of the V3 loop. This structure is key for viral tropism (Cocchi et al., 1996; Wu et al., 1996; Bieniasz et al., 1997; Speck et al., 1997; Isaka et al., 1999) and for the correct CD4 Env binding as revealed with anti-V3 neutralizing antibodies that abrogate Env-CD4 interaction (Trkola et al., 1996; Valenzuela et al., 1997).

In this study, we confirmed the inefficient functionality of the Envs from LTNP-EC individuals previously described and for a cluster of viruses (Lassen et al., 2009; Casado et al., 2018), but extended to HIV+ individuals controlling viremia which are not clustered by the same transmitted/founder (T/F) virus. Also, a gain of Envs functionality from those of the LTNP individuals to the chronic not controlling individuals was identified. This improvement was detected in every Env characteristic analyzed: fusion, virus transfer, and infectivity. Interestingly, this functional growth of viral Env was associated in this study with length and PNGs increases in the variable loops. This increase was also reported in studies analyzing the susceptibility, neutralization sensitivity, co-receptor binding, host range, and viral phenotype (Curlin et al., 2010). This increase in the V1–V2 length and PNGs has also been detected thorough chronic infections from early to late viral Env sampling like in our work (Curlin et al., 2010). Likewise, in a group of individuals infected with closely related viruses higher PNGs density has been observed in the V1–V5 region of the gp120 during chronic infection compared to those observed during the early acute infection phase (Pollakis et al., 2015). In viruses from the HIV-1 subtype B, it seems that early after viral transmission to a new host, a selection for viral variants with shorter variable regions and a reduced degree of PNGs occurs (Liu et al., 2008). The growth in functionality of the viral characteristics was also correlated with the genetic distance of the sequences to the subtype B ancestor. Genetic variability in the *env* gene has been associated with an increase in viral infectivity and replication capacity (Keele et al., 2008; Quan et al., 2009; Fischer et al., 2010; Roche et al., 2011; Fraser et al., 2014; Dang et al., 2020). These changes could facilitate viral replication by increasing viral fitness that favors the escape from the immune response and ART (Koot et al., 1993; Kitrinos et al., 2003, 2005; Kitchen et al., 2004; Hunt et al., 2006; Duenas-Decamp et al., 2008; Salazar-Gonzalez et al., 2008; Kassaye et al., 2009; Moore et al., 2009; Shi et al., 2010).

It has been reported that in a LTNP-EC patient that followed discontinued ART, the V1 domain of his HIV-1 strain that retained good infectivity and replicative capacity included two additional N-glycosylation sites and was placed in the top 1% of lengths among the 6,112 Env sequences analyzed in the Los Alamos National Laboratory online database (Silver et al., 2019).

Furthermore, it is conceivable that the functional characterization of the inefficient HIV-1 Envs could be significant in the development of a new generation of immunogens. Indeed, attenuated HIV or simian immunodeficiency virus (SIV) vaccines (LAHVs or LASVs) have been postulated as therapeutic vaccine strategies (Murphey-Corb, 1997; Desrosiers, 1998; Almond and Stott, 1999; Baba et al., 1999; Johnson, 1999; Mills et al., 2000; Blower et al., 2001). However, further antigenic and immunogenicity work is needed to disclose the potential implications of these non-functional HIV Envs in the vaccine/cure field.

In summary, in this work, we exposed that the characteristics of the viral Envs from different groups of HIV-1 infected individuals could be associated with the short- or long-term VL control and the clinical progression rate of the infection. We observed that Envs from LTNPs exhibited non-functional characteristics in comparison with those from viruses of the progressive infection groups, supporting that the properties of the Envs directly condition HIV-1 infection. Thus, HIV+ LTNP individuals (ECs and Viremics) that exhibit viral control and low clinical progression rates present virus with Env having poor viral functions, whereas functional Envs are from viruses linked with HIV+ patients lacking viral control and presenting clinical progression.

Therefore, viral control could account for an interplay between virus infectiveness and immune response which is conceivable to be more efficient against viruses that bear non-functional Envs, showing poor fusion and infection capacities and very limited evolution, characteristics that we observed in viral Envs from LTNP phenotypes, as shown recently (Lian et al., 2021).

Our data support the hypothesis that the functionality of viral Envs is a key characteristic for the control of viral infection, replication, and pathogenesis. These non-functional HIV-1 Envs could help in the development of new strategies for functional cure and virus eradication.

## Data Availability Statement

The original contributions presented in the study are included in the article/Supplementary Material, further inquiries can be directed to the corresponding authors.

## Author Contributions

SP-Y wrote the paper and performed the research and supervised experimental design. MP and SM performed the research and supervised experimental design. RC-R, VU, RO, CR, JE-H, and IO performed the research. AV-F, CL-G, JB, and CC wrote the paper, conceived and designed the research and laboratory experiments, and supervised experimental design, analysis, and interpretation of data. All authors contributed to the article and approved the submitted version.

## Funding

This work is supported by Spanish AIDS network “Red Temática Cooperativa de Investigación en SIDA” RD12/0017/0002, RD12/0017/0028, RD12/0017/0034, RD16/0025/0011, RDCIII16/0002/0005, and RD16/0025/0041 as part of the Plan Nacional R + D + I and cofunded by Spanish “Instituto de Salud Carlos III (ISCIII)-Subdirección General de Evaluación y el Fondo Europeo de Desarrollo Regional (FEDER).” JB is a researcher from “Fundació Institut de Recerca en Ciències de la Salut Germans Trias i Pujol” supported by the Health Department of the Catalan Government/Generalitat de Catalunya and ISCIII grant numbers PI17/01318 and PI20/00093 (to JB). Work in CL-G and CC lab was supported by grants SAF (2010-17226) and (2016-77894-R) from MINECO (Spain) and FIS (PI 13/02269 and PI20/00093, ISCIII). AV-F’s Lab is supported by the European Regional Development Fund (ERDF), RTI2018-093747-B-100 (“Ministerio de Ciencia e Innovación,” Spain), “Ministerio de Ciencia, Innovación y Universidades” (Spain), ProID2020010093 (“Agencia Canaria de Investigación, Innovación y Sociedad de la Información” and European Social Fund), UNLL10-3E-783 (ERDF and “Fundación CajaCanarias”) and “SEGAI-ULL.” SP-Y is funded by “Fundación Doctor Manuel Morales” (La Palma, Spain) and “Contrato Predoctoral Ministerio-ULL Formación de Doctores” (2019 Program; “Ministerio de Ciencia, Innovación y Universidades,” Spain). RC-R is funded by RD16/0025/0011 and ProID2020010093 (“Agencia Canaria de Investigación, Innovación y Sociedad de la Información” and European Social Fund). JE-H is funded by the Cabildo Tenerife “Agustin de Betancourt” 2017 Program.

## Conflict of Interest

The authors declare that the research was conducted in the absence of any commercial or financial relationships that could be construed as a potential conflict of interest.

## Publisher’s Note

All claims expressed in this article are solely those of the authors and do not necessarily represent those of their affiliated organizations, or those of the publisher, the editors and the reviewers. Any product that may be evaluated in this article, or claim that may be made by its manufacturer, is not guaranteed or endorsed by the publisher.

## Footnotes

^1^ https://indra.mullins.microbiol.washington.edu/webpssm/
