# Supplementary figures and images for: The Characteristics of the HIV-1 Env Glycoprotein Are Linked With Viral Pathogenesis
Source: Front Microbiol. 2022 Mar 24;13:763039. doi: 10.3389/fmicb.2022.763039 (PMC8988142; doi:10.3389/fmicb.2022.763039)

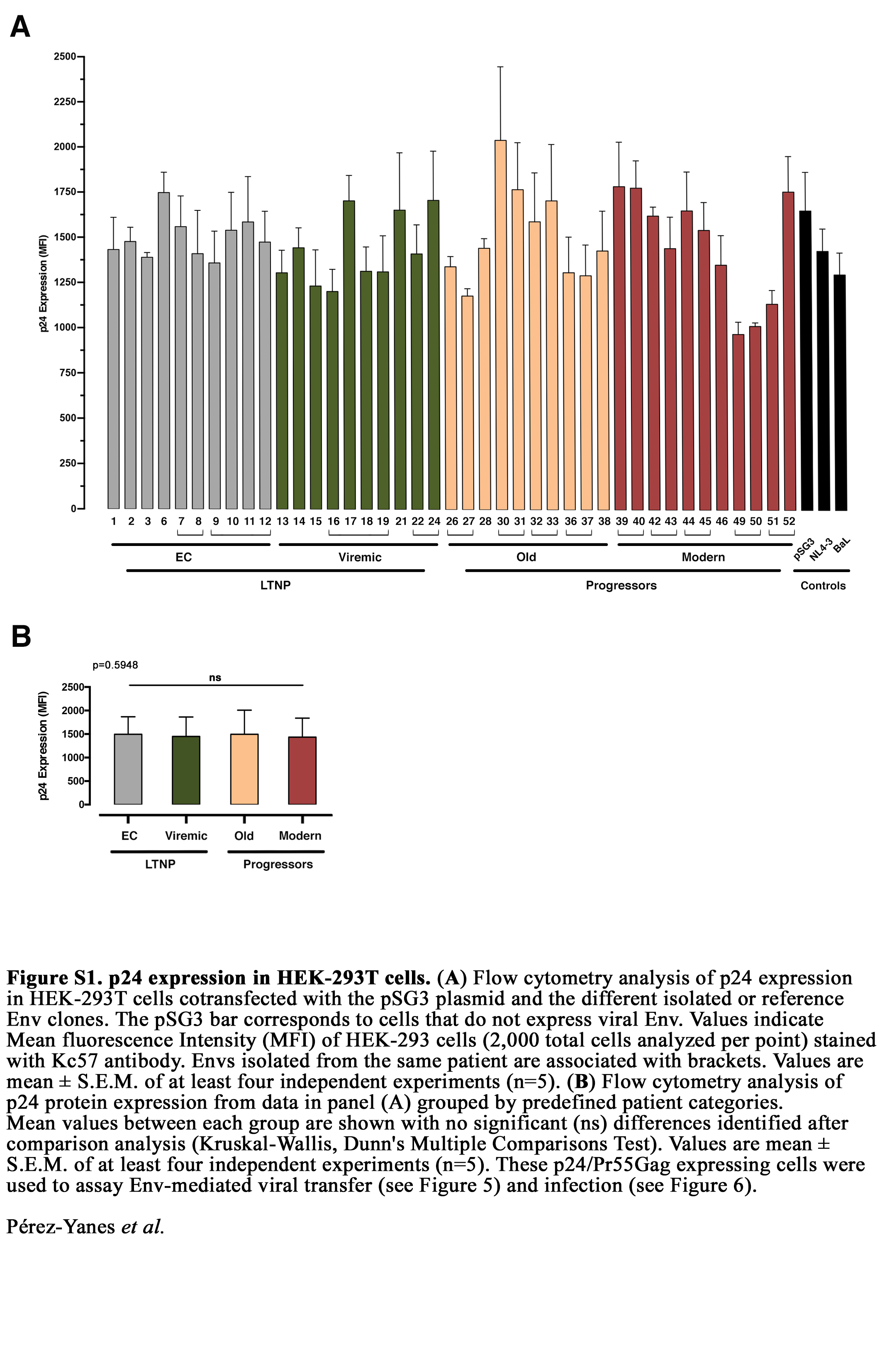

Supplement: Supplementary file 1 [file Image_1.TIF]

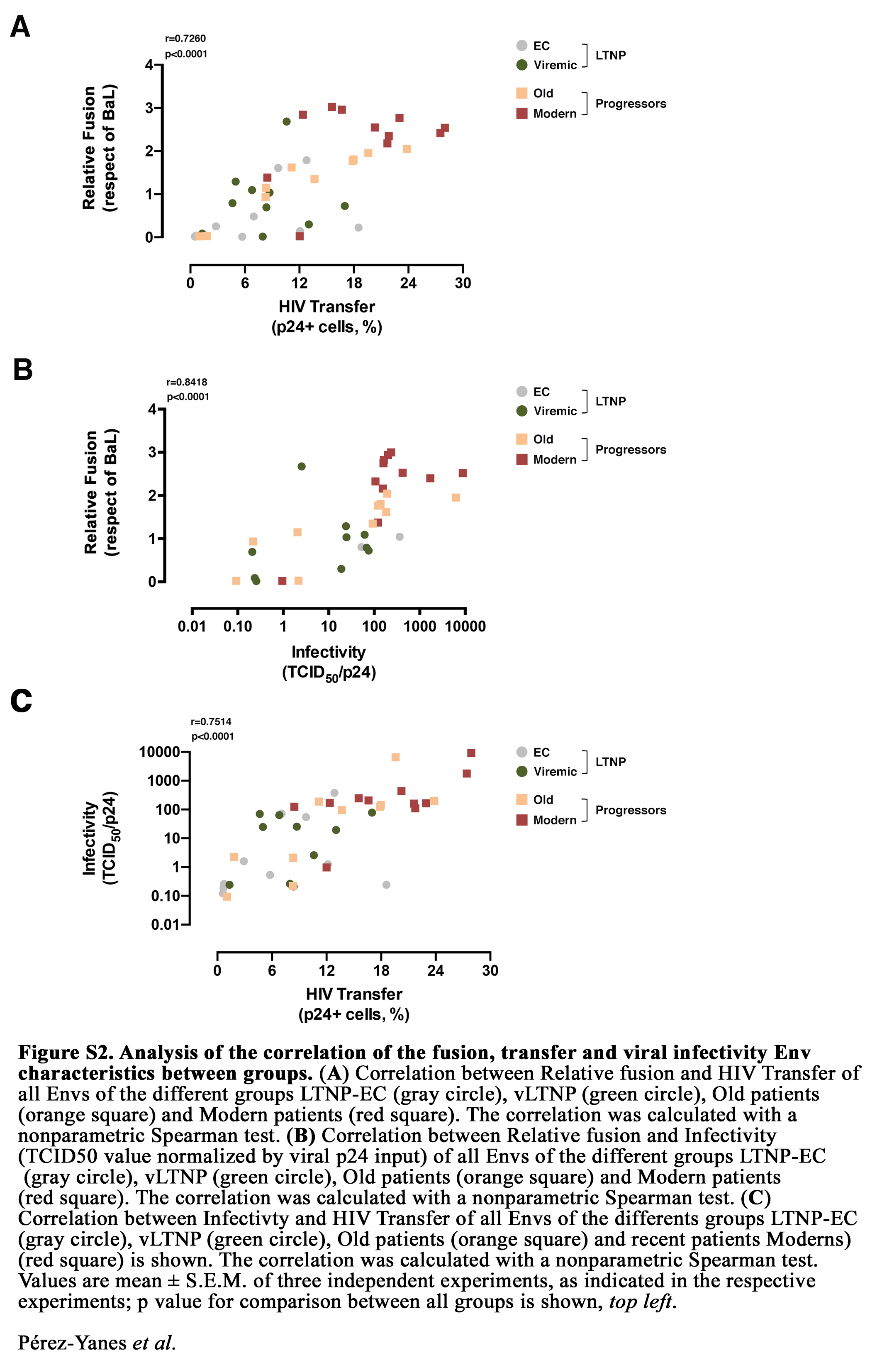

Supplement: Supplementary file 2 [file Image_2.TIF]

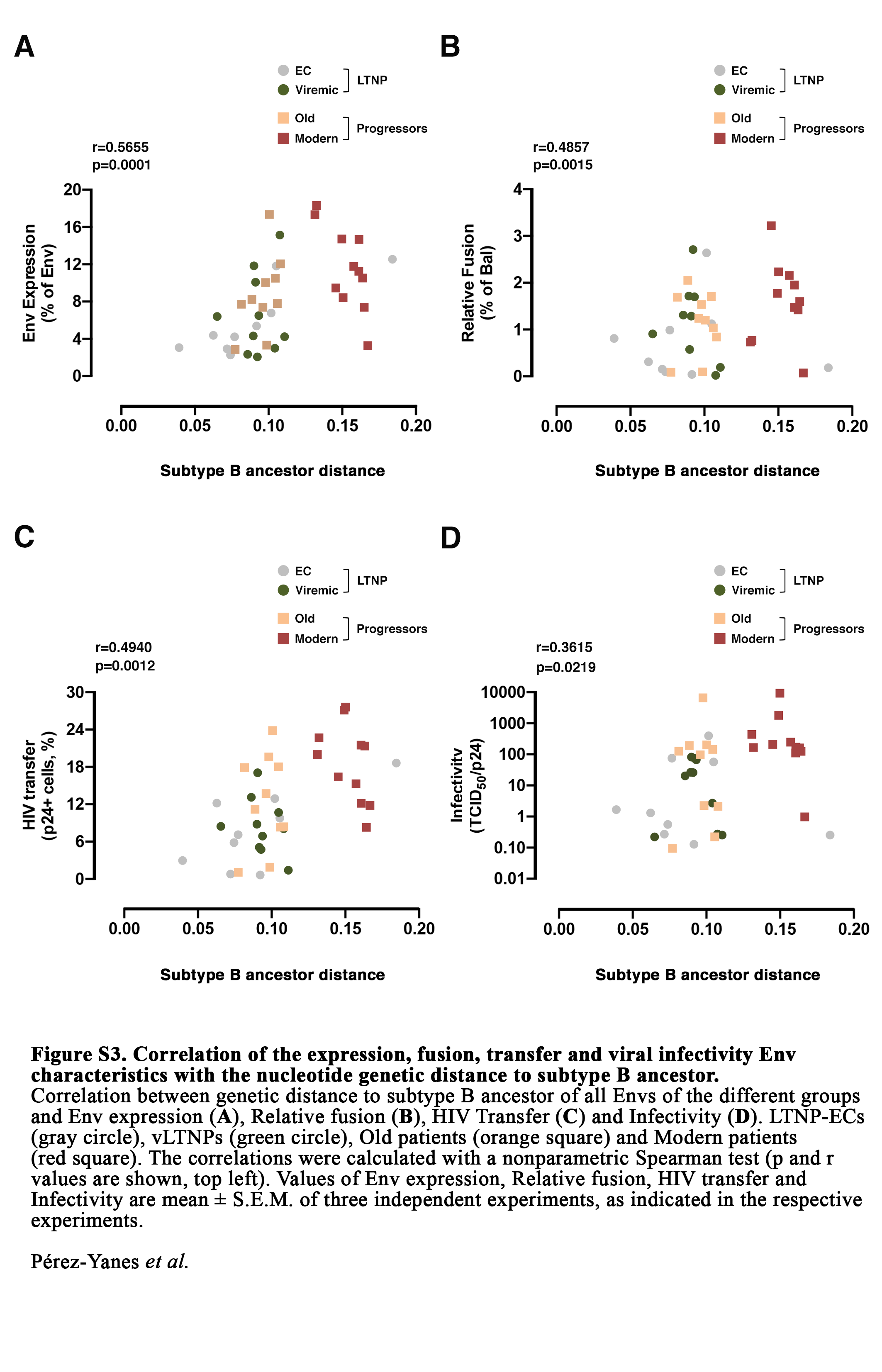

Supplement: Supplementary file 3 [file Image_3.TIF]
